# Supplementary figures and images for: “Two shots for life. HPV-vaccination at your school”. Co-creation of a complex intervention to reduce ethnic inequity in childhood HPV-vaccination in Denmark
Source: PLoS One. 2025 Aug 20;20(8):e0323870. doi: 10.1371/journal.pone.0323870 (PMC12367114; doi:10.1371/journal.pone.0323870)

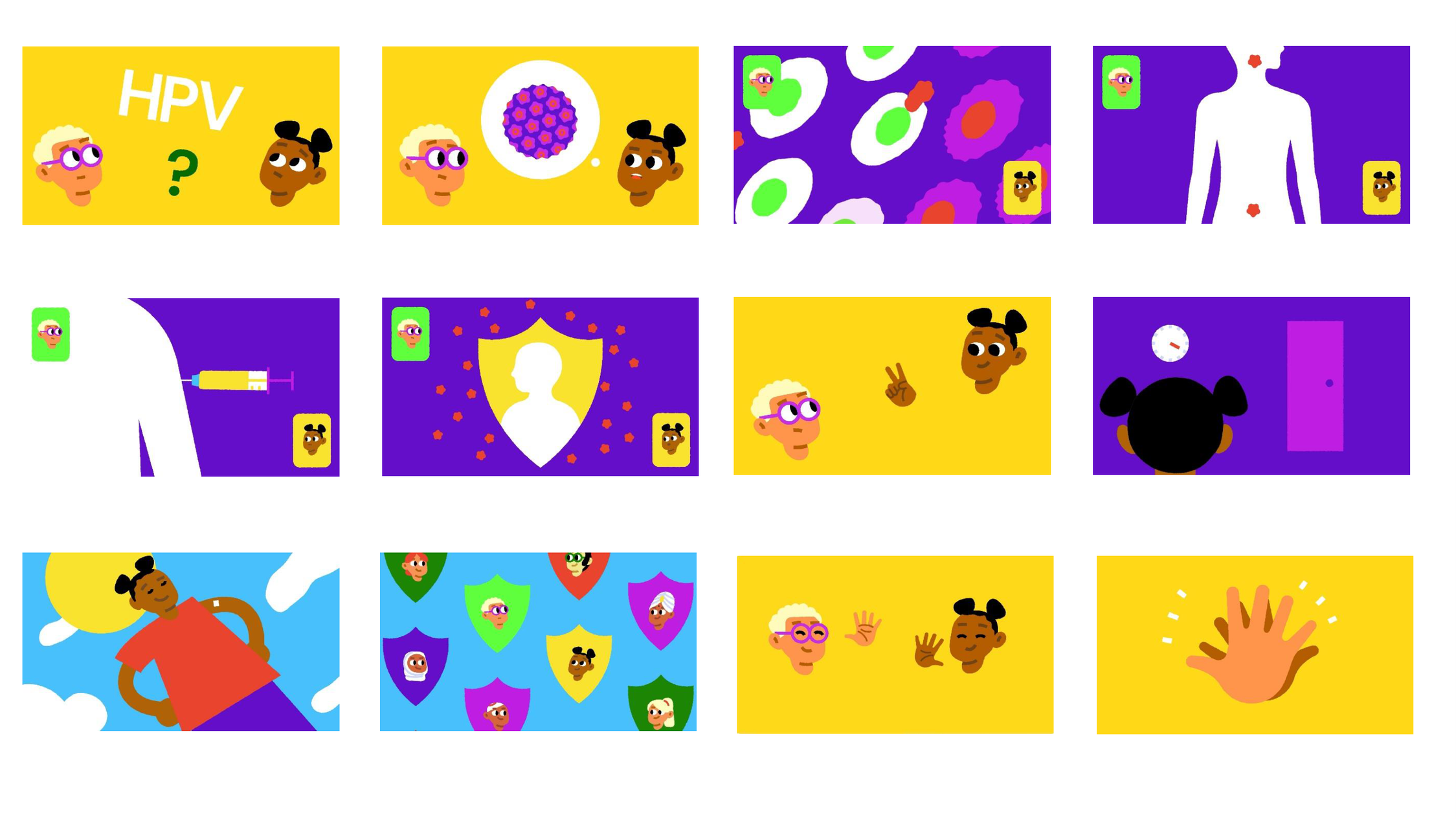

Supplement: S1 File — (TIF) [file pone.0323870.s001.tif]
