# Supplementary material for: “Two shots for life. HPV-vaccination at your school”. Co-creation of a complex intervention to reduce ethnic inequity in childhood HPV-vaccination in Denmark
Source: PLoS One. 2025 Aug 20;20(8):e0323870. doi: 10.1371/journal.pone.0323870 (PMC12367114; doi:10.1371/journal.pone.0323870)
Supplement: Attachment — Submitted filename: Response to reviewers.docx [file pone.0323870.s003.docx]

Dear Academic Editor Maria Lina Tornesello,

Thank you for the opportunity to revise and resubmit our manuscript. We appreciate the comments and suggestions, and we have made changes to the manuscript accordingly.

Below you will find a point-by-point response to the reviewers' comments. First column is copies of the reviewers’ comments; our answers are in second column.

We hope that you find our changes satisfactory. Please do not hesitate to contact us in case of clarifying questions.

We confirm that our submission contains all raw data required to replicate the results of your study. The manuscript is not based on numeric data.

A marked-up copy of the manuscript with tracked changes has been uploaded as well as an unmarked version without tracked changes.

We look forward to hearing from you.

Kind regards,

Anne Katrine Leonhard
*On behalf of all authors*

| This is a well written manuscript describing the development of a school-based complex intervention to promote HPV vaccination to minority families in Aarhus, Denmark. The work takes into account the relevant guidelines and demonstrates this explicitly. One major weakness of the report is that (as found in other comparable projects), one has the impression that the evidence-based justification and development of the format (=> accessibility) is more important than the actual content (=> acceptability). I ignore whether this is a problem with the actual project or only the report – in any case, I suggest that the authors strengthen the content side. | Thank you! We have strived to give a thorough depiction of the development process to give transparency.  In the revised manuscript, we have tried to strengthen the content side by describing more content in the written manuscript (in results from phase 4, especially). Initially, we thought of avoiding double-text, as TIDier is included to give a detailed description of the developed intervention, as is preferred in such reports. Further, illustration examples are attached as Supplement. |
| --- | --- |
| L55: please state to which regions/countries this statement about inequalities applies to. | We have now added countries from referred articles; “…it is a tendency in Denmark and other western countries, that …” |
| L69-72: Please revise the sentence to be more precise: which elements have not yet been addressed in studies and will be addressed here? | Thank you for having a clear eye on our wording here. We have now made small changes in the wording, so we refer to our complex intervention as pioneer regarding combining knowledge of barriers for HPV-vaccination applicable for ethnic minority children, HPV-vaccination programmes and HPV-educational packages. |
| L89 and following: Please describe in the setting or other part of the introduction/methods which was the context for this complex intervention: eg, was this interventional research or a routine public health intervention?  How was it justified – ie, was there are specifically low coverage in Aarhus or Denmark, please show coverage estimates to support the need for targeted action.  Was the intervention supposed to be rolled out over Denmark, following this development and evaluation?  Also, please describe the profile of the minority population in Aarhus, demographically and among participants of the co-construction. | Thank you for this comment.  We have added that the intervention was part of interventional research originated in Aarhus.  The project was justified by lower vaccination coverage among ethnic minorities in DK (specified in L55-56). HPV-vaccination coverage by demographic profiles is known for Denmark, but not for the municipality. Given the size of Denmark we hypothesis that the national coverage (referred to in references), are presentable for Aarhus. We have further added that the ethnic minority population in Aarhus is dominated by non-western origin. Demographic profiles of stakeholders are added in L164-165.  Being the second largest municipality in Denmark, Aarhus is suitable for intervention development aiming to go across sectors and testing whether such intervention could be suitable for upscaling, as described under “implementation and analysis”. |

| L181: The authors describe in the methods that the pre-existing literature concerned cervical cancer prevention in minority women in Denmark. This is only partially relevant for the field of gender-neutral HPV vaccine promotion for minority adolescents. I would therefor recommend developing in more detail the results of the evidence basis, as they were obtained through the specific actions of this project. They should be presented in more structured way and separating elements on adolescents, mothers, fathers … ; on accessibility and acceptability. | Thank you for this comment.  We have rewritten from cervical cancer prevention to HPV-vaccination in L151 since we focused on HPV-vaccination and not cervical cancer screening. We see how this was not clear in the previous written text.  Since boys only have been offered governmental HPV-vaccination since 2019, no demographically statistics are yet available. We have added this limitation in L 422-427. |
| --- | --- |
| As acceptability among minority families does not appear as a main strategic point in this work, please justify more clearly that acceptability was not a main barrier in the targeted population. If this is not the case, both methods and results would need to be revised to explain the underlying concepts (eg, 5C or 7C models are standard nowadays) and intervention levers (use of nudge, any consideration for frequent misconceptions) that were used to address low acceptability. For example: families often think that the child is too young to be vaccinated. If this is the case in the Aarhus minority population, as well, what specific strategy was used to avoid this perception being a barrier to vaccination? | Thank you for this perspective. Acceptability among minority families were a main strategic point in this work; this was the main reason to onboard local community and having the evidence basis being originated from interviews with ethnic minority women representing families in the community. However, we did not onboard any strategic/marketing stakeholder and we therefore don’t have the expertise to go into 5C and 7C models.  In our comprehension, acceptability happens when overcoming mentioned barriers exploited in the result section’s phase 1. In our theoretical framework, we lean upon theories that support a multi-level approach to encounter every layer of potential impact, and hence increase acceptability of the developed intervention. We have added “The joined theoretical framework guided development process in order to achieve a high acceptability of developed intervention.” in L135-137.  We have revisited the manuscript and made it more explicit how we addressed acceptability throughout. |
| Similarly, L204: the authors mention the need to reduce misconceptions, but this neither misconceptions nor barriers to vaccine acceptance are mentioned in phase 1. Please address these points more explicitly. | Misconception was part of the uncertainty and unfamiliarity that came forward in phase 1. We see that we need to explicit this and have added “misconception” in L 198. |
| L201 and following: Acceptability of vaccination could be a main barrier. e | Thank you.  The title of phase 2 “Acceptability of intervention”, refers to onboarding and collaboration with stakeholders, hence having those integrated in development process and by this developing an intervention that implicit is acceptable by all parties involved. We have added this to L87. |
| L231 and following: the authors write that they hypothised that not speaking about HPV as an STI would be more effective. This is a quite weak basis for a key element of the communication strategy, which even is mentioned in the conclusion (L446), in the absence of any evaluation. Please develop the justification for this hypothesis! | Thank you for noticing this poor choice of wording.  The hypothesis was created from previous phases; evidence basis and stakeholder contribution. We have added this detail to L244. |
| L271: Please summarise the key points in which the final material was substantially different from standard material that is used in Denmark for information on HPV vaccination to adolescents. I have the impression that this difference is simpler language and use of minority languages, no mention of STI, and representation of adolescents from different minorities. This impression may be simplistic, so please provide the summary. | Good point, thank you.  We have added a clear summary in L290-2296. |
| L300-326 should be moved further up, as this information was missing in the beginning to understand the context and scope of the present report. Please describe the role of national, regional and local health and school authorities in commissioning or collaborating in this project. | We agree, that “implementation and analysis” should be moved further up.  Ethical considerations, vaccination consent and security are regarding developed intervention and its protocol. We have added this to the headline.  The role of various collaborating authorities is described under Method phase 2 “acceptability of intervention”. |
| L331: I’m wondering here whether school staff was involved (this is addressed later), and if not, why. | This is addressed in discussion, L442-447. |
| The discussion (L356-410) is lengthy and presents elements that I would have hoped to find in the description of the co-construction (evidence). I suggest reorganising the manuscript to bring to the description of the development the specific relevant elements on how which evidence was used. | Thank you. We know that discussion is lengthy, as in many reports concerning intervention development. We have chosen this approach to make a distinct difference between “results” and “discussion” with references included in discussion. This to clarify the difference between our evidence base, results from stakeholder contribution and co-creation, and finally that our results resonate with other’s scientific work. Further, to secure transparency and potential accommodation by other scientists doing interventional research concerning ethnic minorities, school-based vaccination programs, and HPV-vaccination. However, we have tried to shorten the discussion where we found it suitable and further moved some to the result-section. |
| Please have a revision of the English language of the manuscript. | This has been done. |
